# A Real World Report on Intravenous High-Dose and Non-High-Dose Proton-Pump Inhibitors Therapy in Patients with Endoscopically Treated High-Risk Peptic Ulcer Bleeding

**DOI:** 10.1155/2012/858612

**Published:** 2012-07-11

**Authors:** Lung-Sheng Lu, Sheng-Chieh Lin, Chung-Mou Kuo, Wei-Chen Tai, Po-Lin Tseng, Kuo-Chin Chang, Chung-Huang Kuo, Seng-Kee Chuah

**Affiliations:** Division of Hepato-Gastroenterology, Department of Internal Medicine and Kaohsiung Chang Gung Memorial Hospital, College of Medicine, Chang Gung University, 123 Ta-Pei Road, Niaosung Hsiang, Kaohsiung City 833, Taiwan

## Abstract

*Background and Study Aims*. The optimal dose of intravenous proton-pump inhibitor (PPI) therapy for the prevention of peptic ulcer (PU) rebleeding remains controversial. This study aimed to understand the real world experiences in prescribing high-dose PPI and non-high-dose PPI for preventing rebleeding after endoscopic treatment of high-risk PU. *Patients and Methods*. A total of 220 subjects who received high-dose and non-high-dose pantoprazole for confirmed acute PU bleeding that were successfully treated endoscopically were enrolled. They were divided into rebleeding (*n* = 177) and non-rebleeding groups (*n* = 43). Randomized matching of the treatment-control group was performed. Patients were randomly selected for non-high-dose and high-dose PPI groups (*n* = 44 in each group). *Results*. Univariate analysis showed, significant variables related to rebleeding were female, higher creatinine levels, and higher Rockall scores (≧6). Before case-control matching, the high-dose PPI group had higher creatinine level, higher percentage of shock at presentation, and higher Rockall scores. After randomized treatment-control matching, no statistical differences were observed for rebleeding rates between the high-dose and non-high-dose groups after case-control matching. *Conclusion*. This study suggests that intravenous high-dose pantoprazole may not be superior to non-high-dose regimen in reducing rebleeding in high-risk peptic ulcer bleeding after successful endoscopic therapy.

## 1. Introduction

Patients with high-risk stigmata on endoscopic examination for acute upper gastrointestinal bleeding are at increased risk of recurrent bleeding [1]. Endoscopic hemostasis and continuous infusion intravenous high-dose proton-pump-inhibitor (PPI) have been proven to reduce recurrent bleeding, need for surgery, and length of hospital stay [2, 3]. Furthermore, the recently updated Vienna consensus states that intravenous high-dose PPI therapy after successful endoscopic hemostasis decreases both peptic ulcer (PU) rebleeding and mortality in patients with high-risk stigmata [4]. Despite these recent advances in the pharmacological and endoscopic treatment of acute nonvariceal upper gastrointestinal hemorrhage, the associated mortality remains high at 10% to 14% [5]. Theoretically, inhibiting gastric acid and raising the intragastric pH to 6 or more and maintaining it at that level may promote clot stability, thus decrease the likelihood of rebleeding. This is based on experimental data showing that gastric acid impairs clot formation, promotes platelet disaggregation, and favors fibrinolysis [6]. The continuous i.v. infusion of pantoprazole (80 mg bolus plus 8 mg/h continuous infusion) is able to maintain higher intragastric pH for 84% of the time during monitoring, which is higher than intermittent bolus injection (40 mg every 8 h) or lower-dose continuous infusion (40 mg bolus followed by 4 mg/h infusion) and hence should attain better control of peptic ulcer bleeding [7]. However, recent systemic review and meta-analysis of this regiment have shown inconsistent results and the optimal dosing of PPI in preventing PU rebleeding remains controversial [8–10]. This retrospective case-controlled study was conducted to understand the real world experiences in prescribing high-dose PPI and non-high-dose PPI for preventing rebleeding after endoscopic treatment of high-risk PU.

## 2. Patients and Methods

### 2.1. Patients and Study Design

This is a 2-year retrospective chart review case-control study which began in year 2009. Two hundred and twenty patients with gastric or duodenal ulcers bleeding treated successfully via endoscopy were enrolled into this study. All subjects received intravenous PPIs. We excluded patients with malignant ulcers, upper gastrointestinal bleeding unrelated to peptic ulcer such as angiodysplasia and Mallory-Weiss tear, subjects who lost followup less than the required 30 days for reasons other than mortality, and subjects who were unsuccessfully treated during the first endoscopic hemostasis attempt or received inadequate endoscopic hemostasis therapy for high-risk ulcers such as monotherapy with Bosmin injection alone. This was based on our previous study [11] which emphasized that endoscopic epinephrine injection (EI) monotherapy in patients with high-risk ulcers should be avoided. In current studies, only those patients who received initial hemostasis with epinephrine injection combined with thermal therapy or hemoclips [4], or thermal or clip monotherapy [12] are enrolled. Patient's baseline characteristics, concomitant comorbid diseases (including cardiovascular diseases, stroke, liver cirrhosis, chronic obstructive pulmonary disease, diabetes mellitus, and hypertension), presenting hemoglobin levels, platelet counts, hemodynamic status, use of aspirin, nonsteroidal anti-inflammatory drugs (NSAIDs), warfarin/heparin and PPI prior to endoscopic therapy, were recorded using a predetermined spreadsheet. PU bleeding was defined by endoscopist's diagnosis combined with no other identifiable bleeding cause. Endoscopic findings such as ulcer locations, sizes, difficult treatment sites (lesser curvature of high body; posterior wall of bulb and superior duodenal angle), Forrest grade, Rockall scores [13, 14], and treatment methods were also recorded. The endpoints were rebleeding within 30 days after initial endoscopic hemostasis, requirement for surgical intervention, length of hospital stay and total amount of blood transfusion required, bleeding-related mortality, and all-cause mortality. According to results from medical record, these patients were classified into two groups: subjects without recurrent hemorrhage (*n* = 177) and those recurred (*n* = 43).

### 2.2. Definitions

Patients under non-high-dose PPI treatment were defined as those who received 80 mg pantoprazole bolus and followed by i.v. 80 mg per day, until alimentation was possible, then 40 mg per day orally. High-dose PPI therapy were defined as administering 80 mg pantoprazole i.v. bolus injection, then 8 mg per hour continuous infusion for 3 days, followed by i.v. 80 mg per day. Renal function was evaluated by estimated glomerular filtration rate calculated using the 4-variable Modification of Diet in Renal Disease Study equations and classified according to the K/DOQI Clinical Practice Guidelines for Chronic Kidney Disease [15]. High-risk ulcers were defined as Forrest grade higher or equal to 2b [3]. Rebleeding was defined as new onset of hematemesis, coffee-ground vomitus, or hematochezia, with an increasing pulse rate >110 beats/min and decreasing blood pressure below 90 mmHg after a 24-hour period of stable vital signs and hematocrit following endoscopic treatment [11, 16–18]. Total amount of blood transfusion required was defined as units given to the patients between the time PU bleeding occurred and the day of discharge. Bleeding-related mortality was defined as in-hospital death resulted solely from peptic ulcer bleeding.

### 2.3. Statistical Analysis

The quantitative data were compared using the Student's *t*-test for variables with a normal distribution. Differences between the proportions of categorical data were evaluated with Fisher's exact test when the number of expected subjects was less than five and otherwise with the *χ* ^2^ test. The results are expressed as distributions, absolute frequencies, relative frequencies, medians, and ranges, or means ± SD. A multivariate logistic regression model was used to assess the independent association between rebreeding and non-rebleeding groups. *P* value of <0.05 was considered statistically significant. The Statistical Package for Social Sciences (SPSS15.0, Chicago, USA) for Windows was used to analyze the data.

We employed the nearest neighbor-matching method (NCSS 2007, Kaysville, Utah 84037, USA) to reduce bias in the retrospective study. The matching algorithm was performed to find one matched control in high-dose PPIs group for each in non-high-dose group. The matching variables were stage of CKD, Forrest classification and Rockall score, and female gender. As a result, forty-four patients were randomly selected in each group.

## 3. Result

The difference between the two study groups (non-rebleeding versus rebleeding groups) was insignificant in terms of age, medication history such as NSAIDs, clopidogrel, warfarin, initial hemoglobin level, platelet counts, shock at presentation, percentage of high stigmata ulcers, ulcer size, and time to endoscope (Table 1). Univariate analysis revealed significant differences in the following variables: gender (female: 28.2% versus 48.8%, *P* = 0.010), initial creatinine level (2.0 ± 2.3 mg/dL versus 3.1 ± 3.2 mg/dL, *P* < 0.00), use of aspirin (17.5% versus 2.3%, *P* = 0.011), CKD stage III to V (41.2% versus 60.5%, *P* = 0.013), COPD (3.4% versus 11.6%, *P* = 0.026), Rockall score ≧ 6 (59.3% versus 83.7%, *P* = 0.003), amount of blood transfusion of PRBC (879.9 ± 966.4 mL versus 3220.9 ± 2824.3 mL, *P* < 0.001), surgical requirements (0 versus 4.7%, *P* = 0.004), hospital stay (10.6 ± 12.4 days versus 24.6 ± 18.6 days, *P* < 0.001); and mortality (4.5% versus 20.9%, *P* = 0.001). Multivariate analysis showed that the significant factors were sex, high Rockall score, and serum creatinine level (Table 2).

We divided our subjects into two groups: non-high dose and high dose for analysis (Table 3). There were no significant differences between the two groups (non-high dose versus high dose) in terms of patients' gender, age, initial hemoglobin and platelet, NSAIDs, aspirin, clopidogrel, warfarin use, Rockall score, ulcer pattern of Forrest, time to endoscope, duration of hospital stay, surgical interventions, rebleeding rate, and mortality. Significant variables were initial creatinine level (2.0 ± 2.4 mg/dL versus 2.6 ± 2.82 mg/dL, *P* = 0.018), diabetes (25.3% versus 40.0%, *P* = 0.027), CVA (12.0% versus 22.9%, *P* = 0.038), and shock at presentation (46.0% versus 61.4%, *P* = 0.033). Although the Rockall score was not significant between these two groups, it was higher in trend in the high-dose group (5.9 ± 1.7 versus 6.3 ± 1.5, *P* = 0.106).

To minimize the clinical characteristics difference between non-high-dose and high-dose groups, we created a treatment-control randomized match based on CKD stages, Forrest classifications, and Rockall scores. Fifty-six patients were randomly selected in each group of non-high- dose and high dose for analysis (Table 4). All of them have high-risk ulcers according to Forrest classification. As a result, there were no significant differences between the two groups (non-high dose versus high dose) in all demographic and clinical characteristics such as the rebleeding rate (18.2% versus 15.9%, *P* = 0.777), surgery needed (0 versus 0%, *P* = 1.000), and hospital stay (12.1 ± 17.2 days versus 14.3 ± 13.5 days, *P* = 0.505).

## 4. Discussion

After the randomized treatment-control matching process to minimize possible selection bias between the two treatment groups, current retrospective case-controlled study observed that the continuous high-dose PPI regimen did not appear to be more effective in reducing rebleeding compared to non-high-dose regimen in subjects with high-risk ulcer bleeding after initial endoscopic hemostasis in real world clinical practice (18.2% versus 15.9%) as shown in Table 4. Meta-analysis performed by Wang also found that high-dose PPIs do not further reduce the rates of rebleeding, surgical intervention, or mortality after endoscopic treatment in patients with bleeding peptic ulcer [8]. This is contrary to the recently updated consensus statements on the routine use of the intensive PPI regimen for high-risk ulcer bleeding [4].

The explanation to the high rebleeding rate in the current study (43/220, 19.5%) is possibly due to the inclusion of a higher percentage ulcers with high-risk stigmata (214/220, 97.3%) and patients with more severe comorbidities (Rockall score: Mean ± SD = 6.0 ± 1.6). In real world practice, more physicians may prescribe high-dose intravenous PPIs in more severe patients. This may also explain the higher rebleeding rate in the high-dose group (27.1% versus 16.0%) before case-controlled matching. However, the rebleeding rate were identical after case-controlled matching as shown in Table 4. Although we believe that the evidence from our findings may be supportive of the aforementioned studies regarding the issue that low-dose intravenous PPI dosage may be enough in treating peptic ulcer bleeding, potential bias and the relatively small sample size may hinder the conclusion for the optimal dosing of PPIs for bleeding high risk PU.

The other explanation for the possible lower dosage needed for Taiwanese may be attributed to the metabolism of PPI via the pathway of cytochrome P450 system (CYP), where its influential role was considered substantial in this issue [19]. There are more Caucasians than Asians who belong to homozygous extensive metabolizer (EM) in the distribution of genetic polymorphisms of CYP [20, 21], and the effect to maintain intragastric pH > 6.0 in the EM patients with intravenous pantoprazole is inferior to the non-EM patients owing to the lower plasma concentration [22]. Therefore it is rational that this racial difference could suggest that PPI should have better effect in Taiwanese patients [23, 24].

In our study we observed that CKD stage III to V was the independent risk factor for recurrent bleeding. This is despite the fact that all ESRD subjects received heparin-free dialysis in our hospital. Our findings were consistent with Wu et al. [25] and Cheung et al. [26] who reported that patients with ESRD and advanced chronic kidney disease were at higher risk of peptic ulcer rebleeding. The mechanism for the excessive bleeding in patients with ESRD is still unclear but may be multifactorial [27]. Platelet dysfunction in the form of impaired platelet adhesiveness and altered platelet-vessel-wall interaction is believed to have played an important role [28]. Furthermore this platelet dysfunction is not normalized after dialysis [29, 30]. The female gender in our study had higher rebleeding rate before case-controlled matching. This is probably by chance or perhaps, the study number was not big enough, and we need larger study scale to minimize the bias. However, when we re-analyzed the case-matching between the high-dose and non-high-dose groups, this problem does not exist anymore.

We recognized several limitations in this study. First, this retrospective analysis depended heavily on the completeness of the medical charts. If incomplete chart description of ulcer morphology was encountered, we would review endoscopic images or videos to determine the location and severity of the ulcer involved. Second, the selection bias may exist in high-dose group caused by clinicians' decision on PPI dosage in patients with more severe diseases or with less manageable bleeding ulcers. One of the main purposes of the study was to attempt to minimize selection bias by the randomized treatment-control matching process after controlling the baseline conditions of subjects. Although we observed that the rebleeding rates were identical in high-dose and non-high-dose patients after case-controlled matching, the case number was too small for a solid conclusion.

In conclusion, this study suggests that the effect of intravenous high-dose pantoprazole may not be superior to non-high dose regimen in reducing the occurrence of rebleeding, mortality rate, and surgery needed in patients in high-risk peptic ulcer bleeding after successful endoscopic hemostasis. More large scale prospective studies to clarify the issue are still mandatory. In real world practice, election bias may exist in high-dose group caused by clinicians' decision on PPI dosage in patients with more severe diseases or with less manageable bleeding ulcers.

## Figures and Tables

**Table 1:** Univariate analysis of demographic and clinical characteristics of non-rebleeding and rebleeding patients.

Variables	Non-rebleeding group (*n* = 177)	Rebleeding group (*n* = 43)	*P*-value
Age (years)	63.4 ± 13.7	65.2 ± 13.5	0.941
Female gender, *n* (%)	50 (28.2)	21 (48.8)	0.010^∗^
Creatinine (mg/dL)	2.0 ± 2.3	3.1 ± 3.2	<0.001^∗^
Hb (g/L)	97.8 ± 29.4	83.1 ± 23.4	0.074
Platelet (×10^9^/L)	194.8 ± 84.1	183.4 ± 147.5	0.113
Use of NSAIDs, *n* (%)	12 (6.8)	2 (4.7)	0.608
Use of aspirin, *n* (%)	31 (17.5)	1 (2.3)	0.011^∗^
Use of clopidogrel, *n* (%)	18 (10.2)	5 (11.6)	0.779
Use of warfarin, *n* (%)	7 (4.0)	3 (7.0)	0.393
Coexisting illness, *n* (%)			
CKD III to V	73 (41.2)	26 (60.5)	0.013^∗^
COPD	6 (3.4)	5 (11.6)	0.026^∗^
CAD	29 (16.4)	8 (18.6)	0.727
DM	48 (27.1)	18 (41.9)	0.058
CVA	26 (14.7)	8 (18.6)	0.524
Liver cirrhosis	32 (18.1)	7 (16.3)	0.782
High stigmata, *n* (%)	173 (97.7)	41 (95.3)	0.388
Forrest classification Ia/Ib/IIa/IIb/IIc/III	9/100/18/45/5/0	5/31/1/5/0/1	
Shock on admission, *n* (%)	89 (50.3)	23 (53.5)	0.706
Rockall score ≧6, *n* (%)	105 (59.3)	36 (83.7)	0.003^∗^
Time to endoscope (h)	14.3 ± 17.5	19.9 ± 20.2	0.129
Hemostasis methods A/B/C/D/E/F	62/48/11/50/2/4	11/14/0/15/2/1	
Ulcer size (cm)	1.0 ± 0.7	0.9 ± 0.6	0.973
Multiple ulcers, *n* (%)	58 (32.8)	18 (41.9)	0.261
PRBC BT (mL)	879.9 ± 966.4	3220.9 ± 2824.3	<0.001^∗^
Surgery, *n* (%)	0	2 (4.7)	0.004^∗^
Hospital stay (days)	10.6 ± 12.4	24.6 ± 18.6	<0.001^∗^
Mortality, *n* (%)	8 (4.5)	9 (20.9)	0.001^∗^
Bleeding related/other causes	1/7	3/6	

PPI: proton-pump inhibitors, Hb: hemoglobin, CKD: chronic kidney disease, NSAID: nonsteroidal anti-inflammatory drug, PPI: proton-pump inhibitor, DM: diabetes mellitus type 2, COPD: chronic obstructive pulmonary disease, CAD: coronary artery disease, CVA: cerebrovascular accident, BT: blood transfusion, PPI: proton pump inhibitor, Hemostasis methods A/B/C/D/E/F: Bosmin plus APC/heat probe=A, APC/heat probe=B, Hemoclip=C, Bosmin plus hemoclip=D, APC/heat probe plus hemoclip=E, APC plus hemoclip plus Bosmin=F, APC: argon plasma coagulation. ^∗^ *P* < 0.05.

**Table 2:** Multivariate analysis for rebleeding and nonbleeding patients.

	Odds ratio	95% CI.	*P* value
Sex	0.408	0.201–0.828	0.013
High Rockall score	3.215	1.324–7.808	0.010
Creatinine	1.119	0.992–1.263	0.066

**Table 3:** Comparison between the non-high-dose and high-dose PPI before case-controlled matching.

Characteristic	Non-high-dose group (*n* = 150)	High-dose group (*n* = 70)	*P*-value
Age (years)	64.1 ± 13.3	62.6 ± 14.4	0.558
Female gender, *n* (%)	105 (70.0)	44 (62.9)	0.291
Creatinine (mg/dL)	2.0 ± 2.4	2.6 ± 2.8	0.018^∗^
Hb (g/L)	96.2 ± 28.2	92.1 ± 30.1	0.438
Platelet (×10^9^/L)	195.2 ± 103.5	186.8 ± 90.4	0.592
Use of NSAIDs, *n* (%)	8 (5.3)	6 (8.6)	0.359
Use of aspirin, *n* (%)	23 (15.3)	9 (12.9)	0.628
Use of clopidogrel, *n* (%)	15 (10.0)	8 (11.4)	0.747
Use of warfarin, *n* (%)	5 (3.3)	5 (7.1)	0.206
Coexisting illness, *n* (%)			
CKD III, IV/V	47/17 (31.3/11.3)	23/12 (32.9/17.1)	0.422
COPD	8 (5.3)	3 (4.3)	0.740
CAD	21 (14.0)	16 (22.6)	0.102
DM	38 (25.3)	28 (40.0)	0.027^∗^
CVA	18 (12.0)	16 (22.9)	0.038^∗^
Liver cirrhosis	26 (17.3)	13 (18.6)	0.321
Forrest classification Ia/Ib/IIa/IIb/IIc/III	11/86/12/35/5/1	3/45/7/15/0/0	0.524
Shock on admission	69 (46.0)	43 (61.4)	0.033^∗^
Rockall score	5.9 ± 1.7	6.3 ± 1.5	0.106
Time to endoscope (hours)	15.9 ± 19.2	14.1 ± 15.8	0.107
Hemostasis methods A/B/C/D/E/F	11/86/12/35/5/1	3/45/7/15/0/0	
PRBC BT (mL)	11101.7 ± 1495.3	1842.9 ± 2185.7	0.196
Multiple ulcers, *n* (%)	50 (38.7)	26 (37.1)	0.580
Rebleeding, *n* (%)	24 (16.0)	19 (27.1)	0.052
Surgery, *n* (%)	1 (0.6)	1 (1.4)	0.579
Hospital stay (days)	11.9 ± 14.9	16.5 ± 14.3	0.343
Mortality, *n* (%)	9 (6.0)	8 (11.4)	0.207
Bleeding related/other causes	3/6	1/7	

Hb: hemoglobin, NSAID: nonsteroidal anti-inflammatory drug, CKD: chronic kidney disease, PPI: proton-pump inhibitor, DM: diabetes mellitus type 2, COPD: chronic obstructive pulmonary disease, CAD: coronary artery disease, CVA: cerebrovascular accident, BT: blood transfusion, PPI: proton pump inhibitor, Hemostasis methods A/B/C/D/E/F: Bosmin plus APC/heat probe=A, APC/heat probe=B, Hemoclip=C, Bosmin plus hemoclip=D, APC/heat probe plus hemoclip=E, APC plus hemoclip plus Bosmin=F, APC: argon plasma coagulation. ^∗^ *P* < 0.05.

**Table 4:** Comparison between the non-high-dose and high-dose PPI after case-controlled matching.

Characteristic	Non-high-dose group (*n* = 44)	High-dose group (*n* = 44)	*P* value
Age (years)	66.2 ± 12.9	61.7 ± 13.8	0.121
Female gender, *n* (%)	11 (25)	12 (27.3)	0.808
Creatinine (mg/dL)	2.3 ± 2.3	2.6 ± 2.8	0.615
Hb (g/L)	93.3 ± 25.3	92.5 ± 28.7	0.897
Platelet (×10^9^/L)	170.4 ± 86.2	189.2 ± 82.1	0.297
Use of NSAIDs, *n* (%)	3 (6.8)	2 (4.5)	0.696
Use of aspirin, *n* (%)	4 (9.1)	5 (11.4)	0.725
Use of clopidogrel, *n* (%)	3 (6.8)	4 (9.1)	0.694
Use of warfarin, *n* (%)	1 (2.3)	2 (4.5)	0.557
Coexisting illness, *n* (%)			
CKD III, IV/V	19/6	13/7	0.410
COPD	1	0	0.315
CAD	6	10	0.269
DM	12	14	0.640
CVA	12	7	0.195
Liver cirrhosis	10	8	0.597
Shock oat presentation	24	28	0.386
Rockall score	6.1 ± 1.4	6.4 ± 1.5	0.387
Time to endoscope (hours)	18.3 ± 23.9	13.6 ± 17.2	0.299
PRBC BT (mL)	1369.3±1496.5	1596.6 ± 1914.0	0.537
Forrest classification Ia/Ib/IIa/IIb/IIc/III	2/28/1/13	1/28/4/11	0.513
Time to oral PPI (days)	4.5 ± 4.4	6.9 ± 4.8	0.016^∗^
Rebleeding, *n* (%)	8 (18.2)	7 (15.9)	0.777
Surgery, *n* (%)	0	0	1.000
Hospital stay (days)	12.1 ± 17.2	14.3 ± 13.5	0.505
Mortality, *n* (%)	5 (11.4)	3 (6.8)	0.359
Bleeding related/other causes	3/2	3/0	

Hb: hemoglobin, NSAID: nonsteroidal anti-inflammatory drug, CKD: chronic kidney disease, PPI: proton-pump inhibitor, DM: diabetes mellitus type 2, COPD: chronic obstructive pulmonary disease, CAD: coronary artery disease, CVA: cerebrovascular accident, BT: blood transfusion, PPI: proton-pump inhibitor, Hemostasis methods A/B/C/D/E/F: Bosmin plus APC/heat probe=A, APC/heat probe=B, Hemoclip=C, Bosmin plus hemoclip=D, APC/heat probe plus hemoclip=E, APC plus hemoclip plus Bosmin=F, APC: argon plasma coagulation. ^∗^ *P* < 0.05.
